# Aqua­[2,6-bis­(2-pyridylamino)pyridine]sulfatonickel(II) monohydrate

**DOI:** 10.1107/S1600536808035101

**Published:** 2008-11-08

**Authors:** Lu Lu, Jun Wang, Bin Xie, Li-Ke Zou

**Affiliations:** a Department of Chemistry and Pharmaceutical Engineering, Sichuan University of Science and Engineering, Zigong, Sichuan 643000, People’s Republic of China

## Abstract

The Ni atom in the title complex, [Ni(SO_4_)(C_15_H_13_N_5_)(H_2_O)]·H_2_O, has a distorted trigonal-bipyramidal coordination formed by the tridentate 2,6-bis­(2-pyridylamino)pyridine (tpdaH_2_) ligand, one sulfate and one coordinated water mol­ecule. The tpdaH_2_ ligand is three-coordinated, with the N atom of the central pyridine ring in the equatorial position [Ni—N = 1.9961 (14) Å] and the N atoms of the peripheral pyridine rings in the axial positions [Ni—N = 1.9668 (15) and 1.9895 (15) Å]. The remaining equatorial positions are occupied by the O atoms of the sulfate ligand and the water molecule. The H atoms of both NH groups of the tpdaH_2_ ligand are involved in hydrogen bonds with the O atoms of the uncoordinated water mol­ecule and the sulfate group which link the complex mol­ecules, forming an infinite three-dimensional network.

## Related literature

For the properties of transition metal complexes with polypyridylamine ligands, see: Wang *et al.* (1999 ▶). For the tri-pyridyldiamine ligand, see: Jing *et al.* (2000 ▶). For metal–metal inter­actions, see: Cotton *et al.* (1998 ▶); Yang *et al.* (1997 ▶).
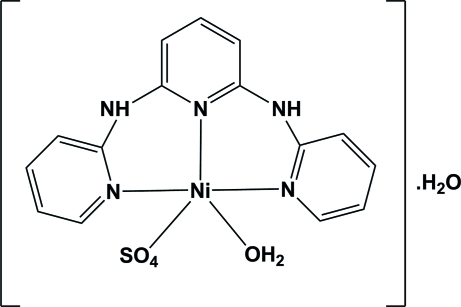


## Experimental

### 

#### Crystal data

[Ni(SO_4_)(C_15_H_13_N_5_)(H_2_O)]·H_2_O
*M* *_r_* = 454.11
Monoclinic, 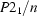

*a* = 7.3536 (8) Å
*b* = 18.026 (2) Å
*c* = 12.9125 (14) Å
β = 95.634 (2)°
*V* = 1703.3 (3) Å^3^
*Z* = 4
Mo *K*α radiation
μ = 1.31 mm^−1^
*T* = 298 (2) K
0.22 × 0.16 × 0.12 mm

#### Data collection

Bruker APEXII area-detector diffractometer
Absorption correction: multi-scan (*SADABS*; Bruker, 2004 ▶) *T*_min_ = 0.761, *T*_max_ = 0.859
8650 measured reflections
3068 independent reflections
2821 reflections with *I* > 2σ(*I*)
*R*_int_ = 0.014

#### Refinement

*R*[*F* ^2^ > 2σ(*F* ^2^)] = 0.021
*wR*(*F* ^2^) = 0.056
*S* = 1.06
3068 reflections
259 parameters
8 restraints
H atoms treated by a mixture of independent and constrained refinement
Δρ_max_ = 0.24 e Å^−3^
Δρ_min_ = −0.32 e Å^−3^

### 

Data collection: *APEX2* (Bruker, 2004 ▶); cell refinement: *APEX2* and *SAINT* (Bruker, 2004 ▶); data reduction: *SAINT*; program(s) used to solve structure: *SHELXS97* (Sheldrick, 2008 ▶); program(s) used to refine structure: *SHELXL97* (Sheldrick, 2008 ▶); molecular graphics: *ORTEPIII* (Burnett & Johnson, 1996 ▶) and *ORTEP-3 for Windows* (Farrugia, 1997 ▶); software used to prepare material for publication: *SHELXL97*.

## Supplementary Material

Crystal structure: contains datablocks I, global. DOI: 10.1107/S1600536808035101/dn2397sup1.cif

Structure factors: contains datablocks I. DOI: 10.1107/S1600536808035101/dn2397Isup2.hkl

Additional supplementary materials:  crystallographic information; 3D view; checkCIF report

## Figures and Tables

**Table 1:** Hydrogen-bond geometry (Å, °)

*D*—H⋯*A*	*D*—H	H⋯*A*	*D*⋯*A*	*D*—H⋯*A*
N2—H2*B*⋯O6^i^	0.866 (9)	2.079 (10)	2.9433 (19)	176 (2)
N4—H4*B*⋯O4^ii^	0.862 (9)	2.050 (10)	2.8967 (18)	167.1 (18)
O5—H5*A*⋯O6	0.86	2.08	2.9112 (18)	163
O5—H5*B*⋯O4^iii^	0.85	1.98	2.8192 (19)	169
O6—H6*A*⋯O2^iv^	0.85	1.91	2.7531 (18)	173
O6—H6*B*⋯O3^iii^	0.85	1.97	2.785 (2)	162

Symmetry codes: (i) 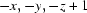; (ii) 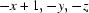; (iii) 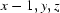; (iv) 
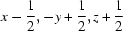
.

**Fig. 1.**
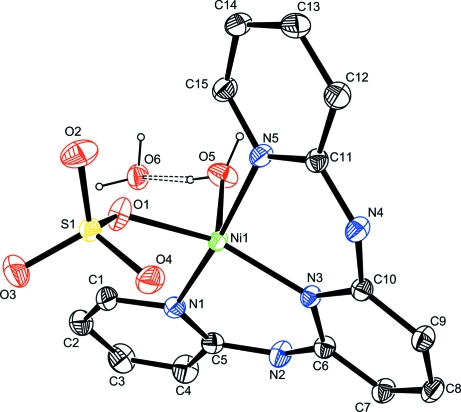
View of compound (I) with the atom-labeling scheme. Displacement ellipsoids are drawn at the 30% probability level. Only H atoms attached to water have been represented as small spheres of arbitrary radii. H bond is shown as dashed line.

## supplementary crystallographic information

### Comment

Transition metal complexes with polypyridylamine ligands, possessing diverse structures and special optical and electromagnetic properties (Wang *et al.*,1999), have aroused great interest among researchers, Tri-pyridyldiamine ligand usually exhibits donor as well as acceptor properties and can be used as a popular chelating ligand (Jing *et al.*, 2000). In recent years great efforts have been taken to synthesize and characterize metal chain complexes which can be used to study the metal-metal interactions (Yang *et al.*, 1997; Cotton *et al.*, 1998). Herein we report the synthesis and crystal structure of the title complex with tri-pyridyldiamine ligand.

The Ni1 atom in the title complex has a distorted trigonal-bipyramidal coordination formed by the tridentate tpdaH_2_ ligand, one sulfate and one coordinated water molecule. (Fig. 1). The tpdaH_2_ ligand is tri-coordinated, with the peripheral N1 and N5 atoms in the axial positions [Ni1—N1 = 1.9895 (15) Å, Ni1—N5 = 1.9668 (15) Å and N1—Ni1—N5 = 169.26 (6)°] and the central N3 atom in the equatorial plane of the bipyramid [Ni1—N3 = 1.9961 (14) Å]. The remaining equatorial positions are occupied by one sulfate and one coordinated water molecule. Selected geometric parameters have been listed in tabel 1.

The three pyridine rings of the tpdaH_2_ ligand are not coplanar. The dihedral angles between the planes of the central pyridine ring and two peripheral rings are 15.0 (7) and 22.7 (3)° respectively. In the title complex the two H atoms of both NH groups of tpdaH_2_ act as active H atoms in forming inter-molecular classical hydrogen bonds (Table 2). The inter-molecular hydrogen bonds function greatly in linking the complex to be a infinite three-dimensional network.

### Experimental

Tripyridyldiamine (0.031 g, 0.12 mmol), NiSO4 (0.26 g, 0.13 mmol), were added in a solvent of acetonitrile, the mixture was heated for six hours under reflux. during the process stirring and influx were required. The resultant was then filtered to give a pure solution which was infiltrated by diethyl ether freely in a closed vessel, three weeks later some single crystals of the size suitable for X-Ray diffraction analysis.

### Refinement

Carbon H atoms were positioned geometrically and treated as riding on their parent atoms, with C—H distances of 0.93Å (pyridine ring) with U_iso_H) 1.2U_eq_(C). The amine H atoms were located in difference maps and freely refined with *U*_iso_(H) 1.2*U*_eq_(N). The water H atoms were located in different map and, in the first stage of refinement,  refined with the O-H and H—H distances restraints to 0.85Å and 1.39Å respectively and with *U*_iso_(H) 1.5*U*_eq_(O). In the last cycle, they were treated as riding on their parent O atoms.

### Crystal data

**Table:**

[Ni(SO_4_)(C_15_H_13_N_5_)(H_2_O)]·H_2_O	*F*_000_ = 936
*M**_r_* = 454.11	*D*_x_ = 1.771 Mg m^−^^3^
Monoclinic, *P*2_1_/*n*	Mo *K*α radiation λ = 0.71073 Å
Hall symbol: -P 2yn	Cell parameters from 3068 reflections
*a* = 7.3536 (8) Å	θ = 2.0–25.2º
*b* = 18.026 (2) Å	µ = 1.31 mm^−^^1^
*c* = 12.9125 (14) Å	*T* = 298 (2) K
β = 95.634 (2)º	Block, green
*V* = 1703.3 (3) Å^3^	0.22 × 0.16 × 0.12 mm
*Z* = 4	

### Data collection

**Table:**

Bruker APEXII area-detector diffractometer	3068 independent reflections
Radiation source: fine-focus sealed tube	2821 reflections with *I* > 2σ(*I*)
Monochromator: graphite	*R*_int_ = 0.014
*T* = 298(2) K	θ_max_ = 25.2º
φ and ω scans	θ_min_ = 2.0º
Absorption correction: multi-scan(SADABS; Bruker, 2004)	*h* = −8→8
*T*_min_ = 0.762, *T*_max_ = 0.859	*k* = −20→21
8650 measured reflections	*l* = −15→13

### Refinement

**Table:**

Refinement on *F*^2^	Secondary atom site location: difference Fourier map
Least-squares matrix: full	Hydrogen site location: inferred from neighbouring sites
*R*[*F*^2^ > 2σ(*F*^2^)] = 0.021	H atoms treated by a mixture of independent and constrained refinement
*wR*(*F*^2^) = 0.056	*w* = 1/[s^2^(*F*_o_^2^) + (0.0291*P*)^2^ + 0.6456*P*] where *P* = (*F*_o_^2^ + 2*F*_c_^2^)/3
*S* = 1.06	(Δ/σ)_max_ < 0.001
3068 reflections	Δρ_max_ = 0.24 e Å^−^^3^
259 parameters	Δρ_min_ = −0.32 e Å^−^^3^
8 restraints	Extinction correction: none
Primary atom site location: structure-invariant direct methods	

### Special details

**Table:**

Geometry. All esds (except the esd in the dihedral angle between two l.s. planes) are estimated using the full covariance matrix. The cell esds are taken into account individually in the estimation of esds in distances, angles and torsion angles; correlations between esds in cell parameters are only used when they are defined by crystal symmetry. An approximate (isotropic) treatment of cell esds is used for estimating esds involving l.s. planes.
Refinement. Refinement of *F*^2^ against ALL reflections. The weighted *R*-factor *wR* and goodness of fit *S* are based on *F*^2^, conventional *R*-factors *R* are based on *F*, with *F* set to zero for negative *F*^2^. The threshold expression of *F*^2^ > σ(*F*^2^) is used only for calculating *R*-factors(gt) *etc*. and is not relevant to the choice of reflections for refinement. *R*-factors based on *F*^2^ are statistically about twice as large as those based on *F*, and *R*- factors based on ALL data will be even larger.

### Fractional atomic coordinates and isotropic or equivalent isotropic displacement parameters (Å^2^)

**Table:**

	*x*	*y*	*z*	*U*_iso_*/*U*_eq_	
Ni1	0.26748 (3)	0.067232 (10)	0.233759 (14)	0.02550 (8)	
N1	0.3089 (2)	0.06540 (7)	0.38831 (11)	0.0315 (3)	
N2	0.2360 (2)	−0.06140 (8)	0.39796 (11)	0.0372 (4)	
H2B	0.215 (3)	−0.0957 (9)	0.4422 (13)	0.045*	
N3	0.28575 (18)	−0.04276 (7)	0.22027 (10)	0.0263 (3)	
N4	0.3172 (2)	−0.03370 (8)	0.03833 (10)	0.0308 (3)	
H4B	0.348 (3)	−0.0593 (9)	−0.0137 (11)	0.037*	
N5	0.1828 (2)	0.07800 (7)	0.08526 (11)	0.0302 (3)	
C1	0.3337 (3)	0.13148 (10)	0.43903 (14)	0.0397 (4)	
H1	0.3680	0.1724	0.4015	0.048*	
C2	0.3110 (3)	0.14091 (11)	0.54147 (15)	0.0464 (5)	
H2	0.3247	0.1874	0.5725	0.056*	
C3	0.2670 (3)	0.07959 (13)	0.59813 (15)	0.0525 (5)	
H3	0.2530	0.0842	0.6687	0.063*	
C4	0.2442 (3)	0.01220 (12)	0.55020 (14)	0.0468 (5)	
H4	0.2156	−0.0295	0.5878	0.056*	
C5	0.2643 (2)	0.00678 (9)	0.44353 (13)	0.0314 (4)	
C6	0.2794 (2)	−0.08911 (9)	0.30303 (12)	0.0288 (3)	
C7	0.3110 (3)	−0.16439 (9)	0.29762 (13)	0.0355 (4)	
H7	0.2963	−0.1949	0.3543	0.043*	
C8	0.3645 (3)	−0.19360 (9)	0.20725 (14)	0.0362 (4)	
H8	0.3949	−0.2436	0.2037	0.043*	
C9	0.3728 (2)	−0.14842 (9)	0.12190 (13)	0.0327 (4)	
H9	0.4099	−0.1672	0.0603	0.039*	
C10	0.3249 (2)	−0.07446 (8)	0.12950 (12)	0.0268 (3)	
C11	0.2342 (2)	0.03302 (9)	0.01132 (12)	0.0274 (3)	
C12	0.2087 (3)	0.05246 (10)	−0.09421 (13)	0.0353 (4)	
H12	0.2458	0.0206	−0.1448	0.042*	
C13	0.1283 (3)	0.11901 (10)	−0.12178 (14)	0.0389 (4)	
H13	0.1129	0.1334	−0.1913	0.047*	
C14	0.0701 (2)	0.16493 (10)	−0.04534 (14)	0.0370 (4)	
H14	0.0126	0.2098	−0.0628	0.044*	
C15	0.0987 (2)	0.14286 (9)	0.05574 (14)	0.0351 (4)	
H15	0.0590	0.1735	0.1069	0.042*	
S1	0.55296 (6)	0.18214 (2)	0.18169 (3)	0.02779 (10)	
O1	0.37943 (18)	0.16682 (6)	0.22894 (10)	0.0409 (3)	
O2	0.5111 (2)	0.22657 (8)	0.08910 (10)	0.0496 (4)	
O3	0.6762 (2)	0.22117 (8)	0.25801 (11)	0.0553 (4)	
O4	0.63271 (18)	0.11012 (7)	0.15417 (10)	0.0417 (3)	
O5	−0.02550 (18)	0.09435 (8)	0.27157 (10)	0.0466 (3)	
H5A	−0.0394	0.1213	0.3252	0.070*	
H5B	−0.1208	0.1008	0.2292	0.070*	
O6	−0.1511 (2)	0.17329 (7)	0.44790 (10)	0.0473 (3)	
H6A	−0.0976	0.2059	0.4872	0.071*	
H6B	−0.2151	0.1947	0.3983	0.071*	

### Atomic displacement parameters (Å^2^)

**Table:**

	*U*^11^	*U*^22^	*U*^33^	*U*^12^	*U*^13^	*U*^23^
Ni1	0.03692 (13)	0.01854 (12)	0.02107 (12)	0.00086 (8)	0.00302 (8)	−0.00001 (7)
N1	0.0354 (8)	0.0303 (8)	0.0283 (7)	0.0020 (6)	−0.0002 (6)	−0.0009 (6)
N2	0.0549 (10)	0.0298 (8)	0.0288 (8)	−0.0026 (7)	0.0136 (7)	0.0031 (6)
N3	0.0305 (7)	0.0233 (7)	0.0256 (7)	−0.0004 (5)	0.0051 (5)	0.0005 (5)
N4	0.0429 (9)	0.0260 (7)	0.0247 (7)	0.0043 (6)	0.0097 (6)	0.0005 (6)
N5	0.0371 (8)	0.0250 (7)	0.0289 (7)	0.0033 (6)	0.0048 (6)	0.0019 (6)
C1	0.0503 (11)	0.0323 (9)	0.0346 (9)	0.0003 (8)	−0.0053 (8)	−0.0044 (7)
C2	0.0556 (13)	0.0447 (11)	0.0373 (10)	0.0055 (9)	−0.0041 (9)	−0.0141 (9)
C3	0.0634 (14)	0.0641 (14)	0.0313 (10)	0.0005 (11)	0.0108 (9)	−0.0123 (10)
C4	0.0602 (13)	0.0507 (12)	0.0314 (10)	−0.0041 (10)	0.0140 (9)	0.0006 (8)
C5	0.0315 (9)	0.0335 (9)	0.0294 (8)	0.0029 (7)	0.0039 (7)	0.0002 (7)
C6	0.0316 (9)	0.0278 (8)	0.0275 (8)	−0.0018 (7)	0.0054 (6)	0.0018 (7)
C7	0.0469 (11)	0.0272 (9)	0.0327 (9)	−0.0019 (7)	0.0051 (8)	0.0063 (7)
C8	0.0451 (11)	0.0221 (8)	0.0413 (10)	0.0027 (7)	0.0032 (8)	0.0010 (7)
C9	0.0392 (10)	0.0270 (8)	0.0327 (9)	0.0022 (7)	0.0071 (7)	−0.0024 (7)
C10	0.0266 (8)	0.0259 (8)	0.0280 (8)	−0.0010 (6)	0.0039 (6)	0.0002 (6)
C11	0.0285 (8)	0.0256 (8)	0.0283 (8)	−0.0029 (6)	0.0041 (6)	0.0014 (6)
C12	0.0443 (11)	0.0342 (9)	0.0278 (8)	0.0003 (8)	0.0057 (7)	0.0002 (7)
C13	0.0459 (11)	0.0401 (10)	0.0299 (9)	0.0004 (8)	−0.0001 (8)	0.0079 (8)
C14	0.0385 (10)	0.0299 (9)	0.0414 (10)	0.0032 (7)	−0.0016 (8)	0.0071 (8)
C15	0.0401 (10)	0.0286 (9)	0.0366 (9)	0.0049 (7)	0.0037 (7)	0.0005 (7)
S1	0.0340 (2)	0.0242 (2)	0.02483 (19)	0.00013 (16)	0.00140 (16)	−0.00106 (15)
O1	0.0525 (8)	0.0268 (6)	0.0467 (7)	−0.0059 (5)	0.0217 (6)	−0.0056 (5)
O2	0.0642 (9)	0.0480 (8)	0.0378 (7)	0.0110 (7)	0.0114 (6)	0.0162 (6)
O3	0.0541 (9)	0.0483 (9)	0.0593 (9)	−0.0030 (7)	−0.0153 (7)	−0.0187 (7)
O4	0.0489 (8)	0.0356 (7)	0.0401 (7)	0.0116 (6)	0.0020 (6)	−0.0076 (5)
O5	0.0364 (7)	0.0672 (9)	0.0361 (7)	0.0131 (7)	0.0035 (5)	−0.0052 (6)
O6	0.0628 (9)	0.0392 (7)	0.0391 (7)	−0.0064 (6)	0.0004 (6)	−0.0034 (6)

### Geometric parameters (Å, °)

**Table:**

Ni1—N5	1.9665 (14)	C4—H4	0.9300
Ni1—O1	1.9784 (12)	C6—C7	1.380 (2)
Ni1—N1	1.9892 (14)	C7—C8	1.373 (3)
Ni1—N3	1.9961 (14)	C7—H7	0.9300
Ni1—O5	2.3077 (13)	C8—C9	1.377 (2)
N1—C5	1.334 (2)	C8—H8	0.9300
N1—C1	1.363 (2)	C9—C10	1.385 (2)
N2—C5	1.370 (2)	C9—H9	0.9300
N2—C6	1.389 (2)	C11—C12	1.402 (2)
N2—H2B	0.866 (9)	C12—C13	1.369 (2)
N3—C10	1.360 (2)	C12—H12	0.9300
N3—C6	1.361 (2)	C13—C14	1.388 (3)
N4—C11	1.378 (2)	C13—H13	0.9300
N4—C10	1.384 (2)	C14—C15	1.361 (2)
N4—H4B	0.862 (9)	C14—H14	0.9300
N5—C11	1.335 (2)	C15—H15	0.9300
N5—C15	1.360 (2)	S1—O2	1.4467 (13)
C1—C2	1.360 (3)	S1—O3	1.4531 (14)
C1—H1	0.9300	S1—O4	1.4821 (12)
C2—C3	1.381 (3)	S1—O1	1.4932 (13)
C2—H2	0.9300	O5—H5A	0.8598
C3—C4	1.366 (3)	O5—H5B	0.8532
C3—H3	0.9300	O6—H6A	0.8477
C4—C5	1.403 (2)	O6—H6B	0.8488
			
N5—Ni1—O1	88.43 (6)	N3—C6—N2	120.04 (15)
N5—Ni1—N1	169.25 (6)	C7—C6—N2	116.92 (15)
O1—Ni1—N1	91.34 (6)	C8—C7—C6	118.96 (16)
N5—Ni1—N3	91.75 (5)	C8—C7—H7	120.5
O1—Ni1—N3	150.08 (5)	C6—C7—H7	120.5
N1—Ni1—N3	93.79 (5)	C7—C8—C9	119.51 (16)
N5—Ni1—O5	88.44 (5)	C7—C8—H8	120.2
O1—Ni1—O5	102.39 (5)	C9—C8—H8	120.2
N1—Ni1—O5	81.11 (5)	C8—C9—C10	118.76 (16)
N3—Ni1—O5	107.52 (5)	C8—C9—H9	120.6
C5—N1—C1	117.63 (15)	C10—C9—H9	120.6
C5—N1—Ni1	121.88 (11)	N3—C10—N4	121.05 (14)
C1—N1—Ni1	117.88 (11)	N3—C10—C9	122.84 (15)
C5—N2—C6	131.54 (15)	N4—C10—C9	116.11 (14)
C5—N2—H2B	112.7 (14)	N5—C11—N4	119.90 (14)
C6—N2—H2B	113.3 (14)	N5—C11—C12	121.54 (15)
C10—N3—C6	116.45 (14)	N4—C11—C12	118.55 (15)
C10—N3—Ni1	120.95 (10)	C13—C12—C11	119.02 (16)
C6—N3—Ni1	122.24 (11)	C13—C12—H12	120.5
C11—N4—C10	131.19 (14)	C11—C12—H12	120.5
C11—N4—H4B	114.3 (13)	C12—C13—C14	119.56 (16)
C10—N4—H4B	112.8 (12)	C12—C13—H13	120.2
C11—N5—C15	118.33 (14)	C14—C13—H13	120.2
C11—N5—Ni1	123.55 (11)	C15—C14—C13	118.56 (16)
C15—N5—Ni1	116.74 (11)	C15—C14—H14	120.7
C2—C1—N1	123.59 (18)	C13—C14—H14	120.7
C2—C1—H1	118.2	N5—C15—C14	122.94 (16)
N1—C1—H1	118.2	N5—C15—H15	118.5
C1—C2—C3	118.20 (18)	C14—C15—H15	118.5
C1—C2—H2	120.9	O2—S1—O3	111.12 (9)
C3—C2—H2	120.9	O2—S1—O4	110.11 (8)
C4—C3—C2	119.76 (18)	O3—S1—O4	110.59 (8)
C4—C3—H3	120.1	O2—S1—O1	108.60 (8)
C2—C3—H3	120.1	O3—S1—O1	108.26 (9)
C3—C4—C5	119.10 (19)	O4—S1—O1	108.06 (7)
C3—C4—H4	120.4	S1—O1—Ni1	123.76 (7)
C5—C4—H4	120.4	Ni1—O5—H5A	118.5
N1—C5—N2	121.08 (15)	Ni1—O5—H5B	128.2
N1—C5—C4	121.67 (16)	H5A—O5—H5B	106.5
N2—C5—C4	117.25 (16)	H6A—O6—H6B	109.1
N3—C6—C7	123.03 (15)		

### Hydrogen-bond geometry (Å, °)

**Table:**

*D*—H···*A*	*D*—H	H···*A*	*D*···*A*	*D*—H···*A*
N2—H2B···O6^i^	0.866 (9)	2.079 (10)	2.9433 (19)	176 (2)
N4—H4B···O4^ii^	0.862 (9)	2.050 (10)	2.8967 (18)	167.1 (18)
O5—H5A···O6	0.86	2.08	2.9112 (18)	163
O5—H5B···O4^iii^	0.85	1.98	2.8192 (19)	169
O6—H6A···O2^iv^	0.85	1.91	2.7531 (18)	173
O6—H6B···O3^iii^	0.85	1.97	2.785 (2)	162

Symmetry codes: (i) −*x*, −*y*, −*z*+1; (ii) −*x*+1, −*y*, −*z*; (iii) *x*−1, *y*, *z*; (iv) *x*−1/2, −*y*+1/2, *z*+1/2.
